# Development and validity of a short web-based semi-quantitative Food Frequency Questionnaire applicable in both clinical and research setting: an evolution over time

**DOI:** 10.3389/fnut.2023.1073559

**Published:** 2023-05-17

**Authors:** Joke Verbeke, Tessy Boedt, Christophe Matthys

**Affiliations:** ^1^Clinical and Experimental Endocrinology, Department of Chronic Diseases and Metabolism, KU Leuven, Leuven, Belgium; ^2^Department of Endocrinology, University Hospitals Leuven, Leuven, Belgium

**Keywords:** nutrition assessment, dietary assessment, methodology, reliability, nutrition survey, food intake

## Abstract

**Background:**

Assessing dietary intake is valuable both in clinical practice and in research. In research and in clinical practice, long-term habitual dietary intake is most often of interest. Therefore, a web-based semi-quantitative Food Frequency Questionnaire (FFQ) was developed to measure habitual intake of nutrients and foods.

**Aim:**

This study aimed to assess content validity, convergent validity, and reliability of a 32-item semi-quantitative FFQ for adults.

**Methods:**

A total of three different cohorts of Flemish adults were recruited in the past 10 years. The first cross-sectional validation study took place in 2013, consequently in 2019 and 2021. Content validity was assessed in 2019 through a semi-structured cognitive interview. Convergent validity was assessed by examining mean differences, Wilcoxon signed-rank test, Spearman's correlation coefficients (SCC), and Bland–Altman analysis for energy, nutrient, and food group intake compared with a 3-day food record (FR). Additionally, consumers-only analysis was performed together with cross-classification analysis by assessing the ranking capabilities of the FFQ into quartiles and weighted kappa. Reliability was assessed through the evaluation of SCC and intra-class correlation (ICC) of test–retest assessment of the FFQ.

**Results:**

Spearman's correlation coefficient (SCC) for energy and absolute nutrient intake between the FFQ and the FR ranged from 0.02 to 0.54. Compared with absolute macronutrients, higher SCC was found for the majority of the relative macronutrient intake and most food groups. Bland–Altman plots showed improved agreement and decreasing bias between the FFQ and the FR over time. Misclassification of the FFQ for nutrients was acceptable and decreased over time (7.4, 7.5, and 6.8% in 2013, 2019, and 2021, respectively), but weighted kappa remained mostly fair (κ ≤ 0.20). The reliability of the FFQ was good and improved over time (mean SCC of 0.65 and 0.66 *p* <0.001 in 2013 and 2019).

**Conclusion:**

The short web-based FFQ is an easy, low-cost, and feasible tool with good reliability, low misclassification, and acceptable validity to compare nutrient densities and food group intake at the population level. The measurement of absolute intake remains debatable.

## 1. Introduction

Assessing dietary intake is valuable both in clinical practice and in research. However, measuring an individual's dietary intake accurately is challenging. The dietary intake of an individual is highly variable due to day-to-day variation, seasonal variation, and the context an individual is in at the time of eating (1, 2). To assure high-quality data, measuring dietary intake quantitatively can only be performed for short period of time. However, in research and in clinical practice, the long-term habitual dietary intake is most often of interest. Additionally, dietary intake is multidimensional and can be described by macro- and micronutrient intake, food group consumption, diet quality, or dietary pattern, as a whole. Consequently, diet is a complex exposure variable existing of different components which are all interrelated (2). Nevertheless, accurate measurements of dietary intake are crucial to research the role of diet in relation to diseases (3). Moreover, in clinical practice, evaluating dietary intake allows to assess nutritional status and provide dietary advice as part of the prevention or treatment of disease. Currently, no method is available to measure true dietary intake, and research is relying on methods approximating true dietary intake. For this reason, dietary assessment methods are known to have inherent measurement error (4).

Food Frequency Questionnaires (FFQs) are amongst the most used methods to measure dietary intake in large epidemiological studies (5). While classic food diaries or 24-h recalls measure short-term dietary intake, FFQs assess habitual intake over a longer period of time (5). The length and extensiveness of an FFQ can differ between a couple of questions on specific food items, such as a “screener,” or can cover an extensive list of food items to assess the overall dietary pattern. Typically, an FFQ consists of questions both on the amount and the frequency of consumption of specific foods over a predefined period of time. However, FFQs are often characterized by an extensive enumeration of questions on more than 100 food items resulting in long questionnaires (6). Although widely used in large-scale epidemiological studies and clinical practice, the length of the questionnaire still holds a substantial burden and limits feasibility. Therefore, the aim of this study was to develop a short FFQ, intended to be concise yet accurate.

The self-administered semi-quantitative web-based FFQ was developed in Dutch and allows to measure dietary habits, dietary quality, and habitual intake of foods and nutrients during the past month in Belgian adults in both the clinical setting and nutritional research. This study describes the validation of this specific FFQ during the past decade in different cohorts and the improvement of the FFQ accordingly.

## 2. Materials and methods

This study applied the STROBE-NUT guidelines (7).

### 2.1. Study design and study population

This research is a sequential cross-sectional validation study of a web-based semi-quantitative Food Frequency Questionnaire (FFQ) in which data were collected in 2013, 2019, and 2021 including different cohorts of the Belgian population. Content validity, reliability, and convergent validity compared with a 3-day food record were assessed at the three different time periods. Convergent validity was assessed in all three studies by comparison with a 3-day food record (2013, 2019, and 2021), reliability was assessed by test–retest assessment in the cohorts of 2013 and 2019, and content validity was solely assessed in 2019 in a sample of 15 participants through a semi-cognitive interview.

**Figure 1:**
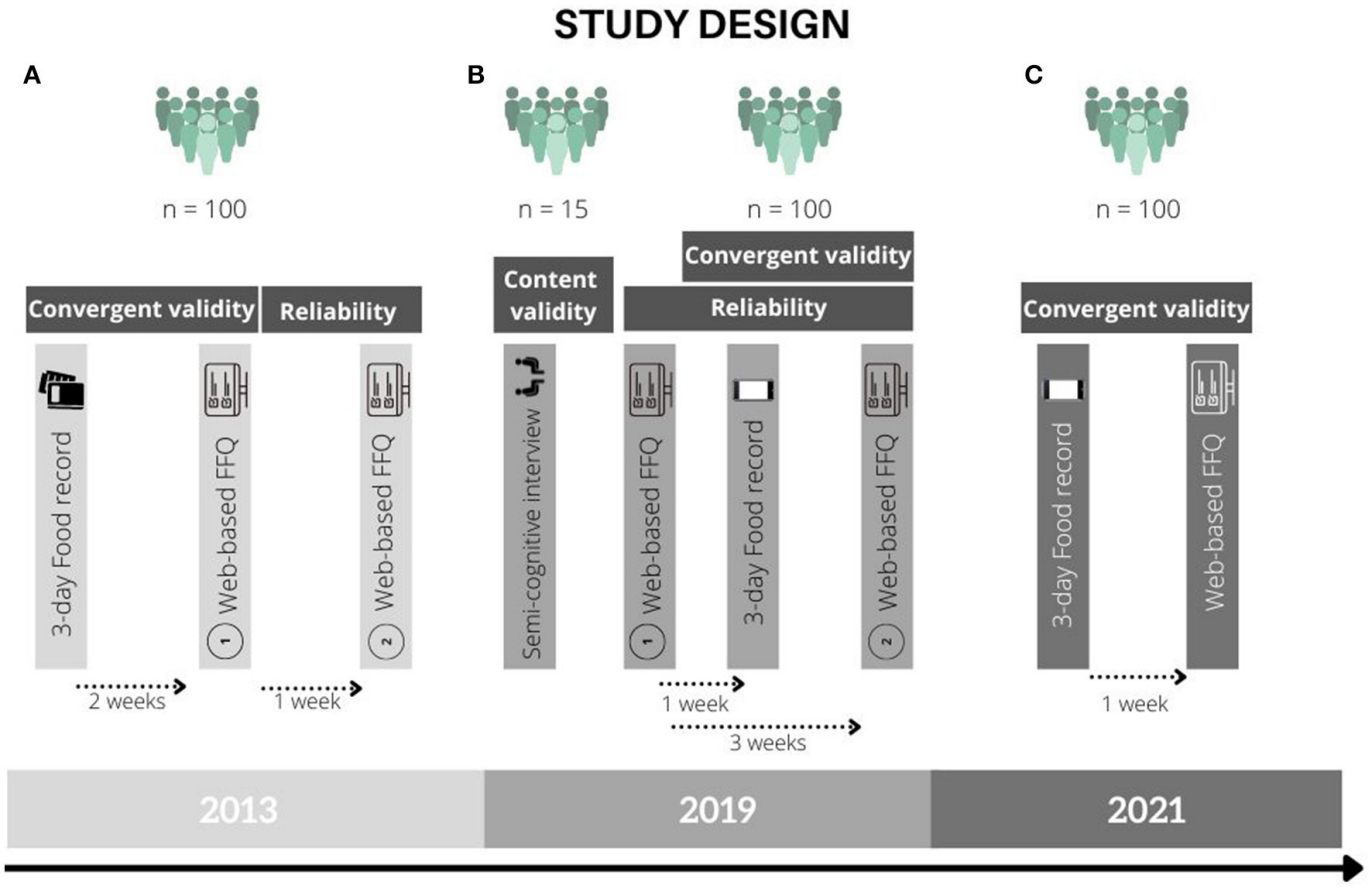
Study design of the validation studies in the years 2013, 2019, and 2021. **(A)** 2013. **(B)** 2019. **(C)** 2021.

The studies were approved by the Medical Ethical Committee UZ Leuven (2013) or the Social and Societal Ethics Committee (SMEC) review board (2019, 2021) and received registration numbers S54908, G-2017 12, and G-2021-2956, respectively. All participants provided their written informed consent prior to the start of the study. The study protocol of the three cohorts in the different time periods during the past decade is shown in Figure 1. Participants in all three cohorts filled out a generic sociodemographic questionnaire at the beginning of the study as well. Participants were allocated an identification number so that the analysis of all questionnaires and food records was anonymised. The different sub-studies took place in Flanders, the Northern Dutch-speaking part of Belgium.

To assess the validity and reliability of the FFQ, a sample size of each time of 100 participants was aimed for. The sample size was based on the recommendations by Cade et al. and Willet et al. stating that a sample size of minimum of 50 participants, preferably at least 100, is sufficient to assess the convergent validity of Food Frequency Questionnaires (5, 8). The cohorts of the validation studies were imbedded in different cross-sectional studies. Therefore, different inclusion criteria were applied to the different cohorts. For the cohort of 2013, participants were recruited by students through word of mouth and by contacting communities through e-mail. Inclusion criteria for the 2013 sample were having an age of 40–70 years old, having no cardiovascular diseases, not following a specific diet for medical reasons, and not having other family members enrolled. The 2019 sample was recruited through calls on social media together with the snowball sampling method. Inclusion criteria for the 2019 sample were being of reproductive age (i.e., 18–42 years old), speaking Dutch, and not following a specific diet for medical reasons. Recruitment of the 2021 cohort took place through word of mouth or calls on social media together with the snowball sampling method. Inclusion criteria were having knowledge of Dutch, not being pregnant, or lactating. Exclusion criteria were individuals living in a country other than Belgium and individuals following a specific diet because of a medical indication including but not limited to persons with Coeliac disease, type 2 diabetes mellitus, food intolerances or allergies and persons who underwent bariatric surgery previously.

### 2.2. Development and design of FFQ

The Food Frequency Questionnaire was developed to acquire standardized data on habitual dietary intake in adults during the past month. It is aimed to use a simple, quick, and low-cost method to assess dietary intake in epidemiological and nutritional studies within the general population in Flanders as well as in a clinical setting, hence its concise format. The FFQ was developed in Dutch (Belgium). The first version of the FFQ consisted of questions on the consumption of 27 food items, where frequency and amount consumed are administered in close-ended questions, based on a previous study (9–14). Table 1 provides an overview of the different food groups that are part of the initial FFQ. The FFQ development was in line with the recommendations of Cade et al. (5). Food items included in the questionnaire were selected based on the Belgian dietary habits and assessed during the 2004 Belgian Food Consumption Survey and the food categories as described in the Flemish Food-Based Dietary Guidelines (15, 16). The frequency of consumption of each food item can be indicated on the FFQ as never, 1 to 3 days per month, 1 day per week, 2 to 4 days per week, 5 to 6 days per week, or every day. The amount of consumption of each food item can be indicated in the multiple-choice answers, where different amounts of the food item are given in grams, milliliters, or pieces, in case of eggs, to choose from. Questions on the amount of consumption usually provide examples of typical amounts of the questioned food item expressed in grams or milliliters, such as “1 teaspoon = 10 grams” or “1 glass = 150 ml,” to reduce the risk of incorrect estimated amounts by the participants. Additional open-ended or close-ended questions on the specific type or brand were included for some of the food items. The average individual daily intake of energy and nutrients was calculated by multiplying the consumed amount and frequency of consumption indicated in the FFQ and the nutrient content per food item. The nutrient content of the different food items was based on the weighted average of the foods included in the food category. The weighted average is based on the consumption data of the 2004 Belgian Food Consumption Survey. The FFQ was first developed in 2012. In 2019, the FFQ was adapted to the newly introduced Flemish Food-Based Dietary Guidelines of 2019. This included the addition of questions on new food group items such as “nuts and seeds,” “legumes,” “oats and wholegrain cereals,” “coffee,” “soup,” “sweetened dairy products,” and “savory snacks” and the elimination of the question on salt intake. This resulted in an FFQ of 33 food items (Table 1).

**Table 1:** Overview of different food groups questioned in the FFQ of 2013 and 2019 onwards.

**Food groups FFQ 2013**	**Food groups FFQ 2019 and 2021**
Water, coffee, and tea	Water and tea
	Coffee
Soda and fruit juice	Soda and fruit juice
Alcoholic beverages	Alcoholic beverages
Full-fat or sweetened dairy products	Full-fat dairy products
	Sweetened dairy products
Skimmed or semi-skimmed dairy products	Skimmed or semi-skimmed dairy products
Fruit	Fruit
Wholegrain bread or buns	Wholegrain bread or buns
White bread or buns	White bread or buns
Breakfast cereals	Cornflakes and cruesli
	Oats and wholegrain cereals
	Nuts and seeds
Fish spread	Fish spread
Deli meats	Deli meats
Cheese	Cheese
Sweet spread	Sweet spread
Eggs	Eggs
Fish, crustaceans and shellfish	Fish, crustaceans, and shellfish
Vegetarian meat substitute	Vegetarian meat substitute
	Legumes
Meat, poultry, and game	Meat, poultry and game
Potatoes	Potatoes
Wholegrain pasta or rice	Wholegrain pasta or rice
White pasta or rice	White pasta or rice
Vegetables	Vegetables
Sauces	Sauces
Sweet and savory snacks	Sweet snacks
	Savory snacks
Fats and oils	Fats and oils
Fried foods	Fried foods
Salt	
Margarine	Margarine
	Soup

### 2.3. Semi-cognitive interview

To assess the content validity of the FFQ, a semi-structured cognitive interview was performed in 2019, as described by Patrick et al. (17, 18) (Figure 1). During the interview, the interviewer went over every question of the questionnaire with the participant while the participants filled in the questions and explained how they interpret the questions and its components in order to evaluate the comprehensibility and understandability of the questions included in the FFQ. To evaluate the content validity of the questions regarding the amount of the consumed food items, participants were requested to point out the amount of the food item that corresponds best with their amount of consumption in a booklet with typical portion sizes of different foods visualized (19). Additionally, participants were asked to select the glass, cup, and/or mug that corresponded with the described quantities given as an example in the questions of the FFQ. Food groups of which portion sizes were evaluated in comparison with pictures of standard portion sizes included “meat,” “fish,” “sauce,” “cooking fat,” “pasta,” “potatoes,” “vegetables,” “legumes,” “beans,” “chick peas,” “meat spread,” “fish spread,” “sweet spreads,” and “nuts and seeds.”

### 2.4. Food record

In all three cohorts, participants were asked to keep a food record for 3 non-consecutive days including 1 day during the weekend (Figure 1). In the cohort in 2013, paper-based food diaries were used. Participants received instructions on the correct registration of all food consumed in the food diaries prior to the study. In the cohorts of 2019 and 2021, participants were requested to keep a food record through the mobile application or web-based version of “MyFitnessPal.” Participants were requested to register the type of food or recipe and amount consumed in the mobile application of all consumed food items over 3 days. Before registration of the dietary record, an instruction booklet was provided to the participants through e-mail, with guidelines on how to use and register their daily dietary intake correctly in “MyFitnessPal.” Daily consumption of energy and nutrients was calculated as the average of the daily nutrient intake for the 3 days registered in the dietary record. Nutrients of the consumed food items were calculated using the Belgian Food Composition Table (NUBEL, fifth edition, 2009) (20). Participants with an average daily energy intake of <500 kcal per day for women or <800 kcal per day for men and >3,500 kcal per day for women or >4,000 kcal per day for men were excluded from the study (21).

### 2.5. Statistical analysis

Only complete FFQs and food diaries consisting of 3 days with at least two main meals (breakfast, lunch, or dinner) registered daily were considered complete and included for analysis. Convergent validity was assessed by comparing mean differences and Spearman's correlation coefficients for energy, macronutrients, micronutrients, and food groups obtained from the FFQ with the mean intake obtained from the food records (FRs). Protein, total fatty acids, saturated fatty acids (SFA), monounsaturated fatty acids (MUFAs), polyunsaturated fatty acids (PUFAs), total carbohydrates, monosaccharides/disaccharides, and polysaccharides were expressed as energy densities (i.e., percentage of total energy intake that the nutrient is accountable for), next to the absolute values, to adjust for energy intake. Similarly, micronutrients were expressed as relative values (i.e., intake per 100 kcal), next to the absolute values. Mean and standard error (SE) for intake of each nutrient and food group measured by the FFQ and the food record were calculated (Supplementary Tables S1, S2). Since correlation coefficients only assess relatedness between the two methods, the Bland–Altman (BA) method was applied, as well, to assess agreement between the two methods (5). Limits of agreement were calculated as mean difference ± 1.96 ^*^ SD. Good agreement was interpreted as at least 95% of the data points lying within the limits of agreement in the BA plot. Reliability was examined by test–retest assessment of two administrations of the FFQ. Spearman's correlation coefficients and intra-class correlation coefficients (ICC) were calculated to assess the reliability of the FFQ, next to a Wilcoxon signed-rank test to examine the statistically significant difference between the mean of the two administrations of the FFQ. ICC was assessed in a two-way mixed-effects model considering absolute agreement for a single measurement, as recommended by Koo et al. for test–retest reliability assessment (22). Next, consumers-only analysis (i.e., taking only consumers for each specific food group into account) was performed similarly to assess convergent validity for each food group (Supplementary Table S3). Finally, cross-classification analysis was performed to assess the percentage of misclassification by the FFQ compared with the 3-day food record. Spearman's correlation coefficients were interpreted according to Cohen's cutoff values, where *r* = ±0.5 was considered to be strong, *r* = ±0.30 moderate, and *r* = ±0.10 weak (23). ICCs were interpreted as ICC ≤ 0.40 is “poor,” 0.41 < ICC < 0.59 is “fair,” 0.60 < ICC < 0.74 is “good,” and 0.75 < ICC < 1.00 is “excellent” (24). A *P*-value of < 0.05 indicated statistical significance. The statistical program IBM SPSS Statistics 27 was used for all analyses.

## 3. Results

### 3.1. Population characteristics

Initially, 111, 114, and 44 participants were recruited in the cohorts of 2013, 2019, and 2021, respectively. Participants with an incomplete or missing FFQ or 3-day food record were excluded from the analysis [*n* = 11 (2013), *n* = 8 (2019), and *n* = 2 (2021)]. As previously described, participants with a self-reported energy intake lower than 500 or 800 kcal per day for women or men, respectively, or higher than 3,500 or 4,000 kcal per day for women and men, respectively, were excluded as well [*n* = 0 (2013 and 2021), *n* = 6 (2019)]. The final samples for analysis included 100, 100, and 42 participants in the years 2013, 2019, and 2021, respectively. Only 37 out of 42 participants of the cohort of 2021 responded to the sociodemographic questionnaire. Characteristics of the study population are shown in Table 2. All three cohorts included more women than men (64.4, 58.0, and 81.1% for 2013, 2019, and 2021, respectively). The sample of the year 2019 included younger participants (mean age 25.8 ± 0.52 (SE) years old), including most students, compared with the participants in the samples of the years 2013 and 2021 (mean age 53.5 ±0.65 (SE) years old and 40.9 ±2.18 (SE), respectively). Mean body mass index (BMI), based on self-reported weight and height, was 25.2 ±0.38 (SD) kg/m^2^ for the study population in 2013 and 23.7 ± 0.46 (SD) kg/m^2^ for the study population in 2021. Length and weight were not reported in the study population of 2019. All three cohorts included proportionally more participants with higher education of at least college or university level degree (54.0, 58.0, and 100.0% for 2013, 2019, and 2021, respectively).

**Table 2:** Population characteristics of validation studies of 2013, 2019, and 2021.

	**2013**		**2019**		**2021**	
**Participants**	***n***	**%**	***n***	**%**	***n***	**%**
Total	100	100.0%	100	100.0%	37	100.0%
Men	35	35.0%	42	42.0%	7	18.9%
Women	65	65.0%	58	58.0%	30	81.1%
**Biometrics**	**Mean**	**SE**	**Mean**	**SE**	**Mean**	**SE**
Age (years)	53.5	0.65	25.8	0.52	40.9	2.2
Length (cm)	169.8	0.86			171.0	1.11
Weight (kg)	72.9	1.38			69.9	1.87
Body mass index (BMI) (kg/m^2^)	25.2	0.38			23.7	0.5
**Educational level**	**n**	**%**	**n**	**%**	**n**	**%**
Secondary educational level	46	46.0	42	42.0	0	0.0
Higher educational level	54	54.0	58	58.0	37	100.0

### 3.2. Content validity

Content validity was assessed in 2019 in a subsample of 15 participants with a mean age of 29 ±1.45 (SE) years old including eight women and seven men. According to this evaluation, correctly estimated portion sizes mentioned in the FFQ by participants ranged from 53.3 to 92.3%. The portion sizes of the food groups “vegetables” and “potatoes” were least often estimated correctly by the participants (estimated correctly in 53.3% of participants for both the food groups). The portion sizes of the food groups “sweet spreads” and “sauce” were most often estimated correctly by 92.3 and 86.7% of the participants, respectively. Overestimation of the portion sizes mentioned in the FFQ was lowest for the food group “sweet spreads” (by 0% of participants) and highest for the food groups “meat” and “vegetables” (both by 33.3% of participants). The portion sizes of the food groups “sauce” and “meat spread” were never underestimated by participants, while the portion sizes of “chick peas,” “fish spread,” and “potatoes” were most often underestimated (by 36.4, 33.3, and 33.3% of participants, respectively). The content validity study disclosed further that specific words used in the phrasing of questions in the FFQ are open to multiple interpretations. Afterwards, the phrasing of questions and usage of words were changed according to the feedback received during the interviews in the content validity study.

### 3.3. Convergent validity

#### 3.3.1. Macronutrients and micronutrients

**Figure 2:**
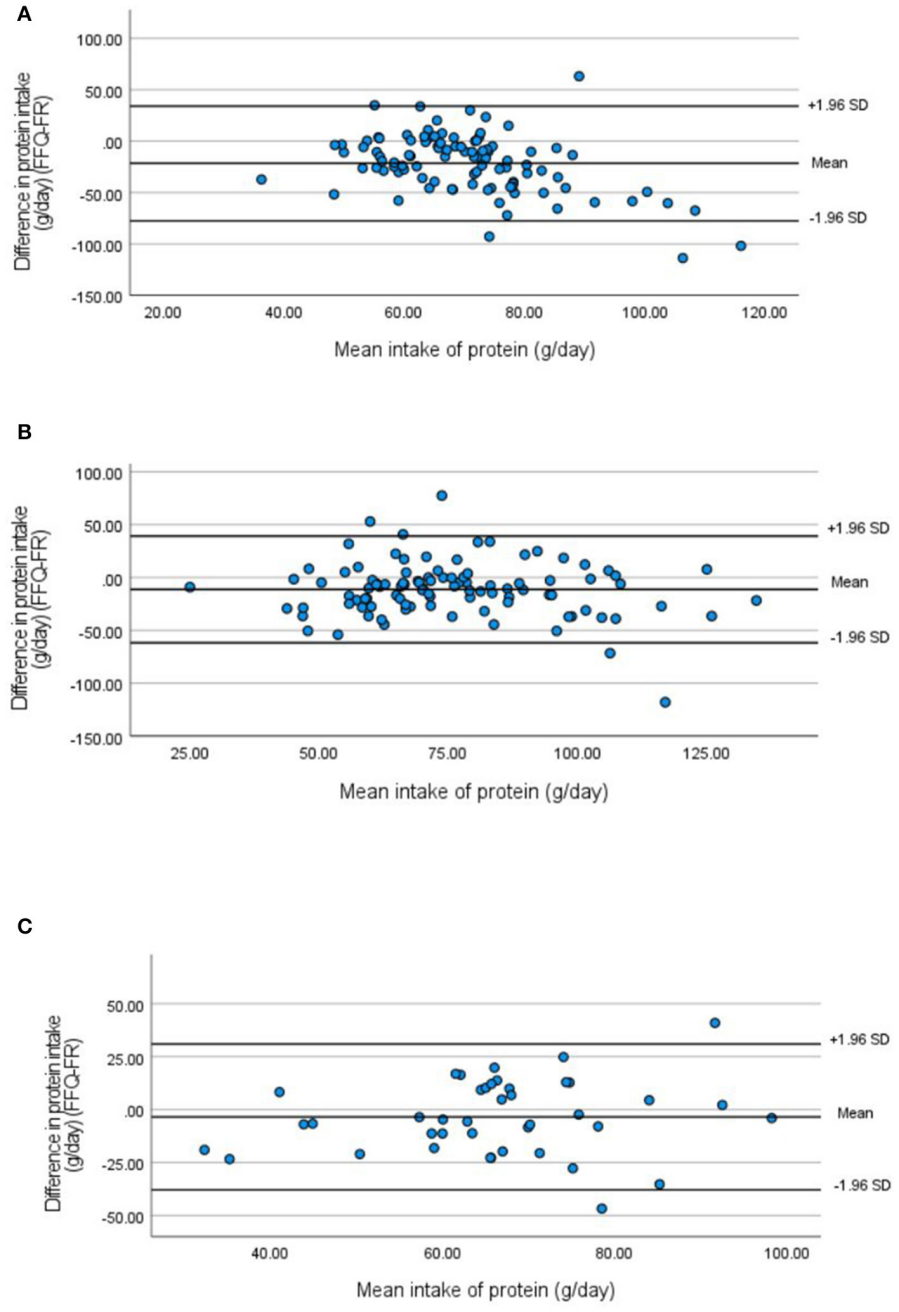
Bland–Altman plots for absolute protein intake measured by the FFQ compared with the 3-day food record in 2013 **(A)**, 2019 **(B)**, and 2021 **(C)**.

**Figure 3:**
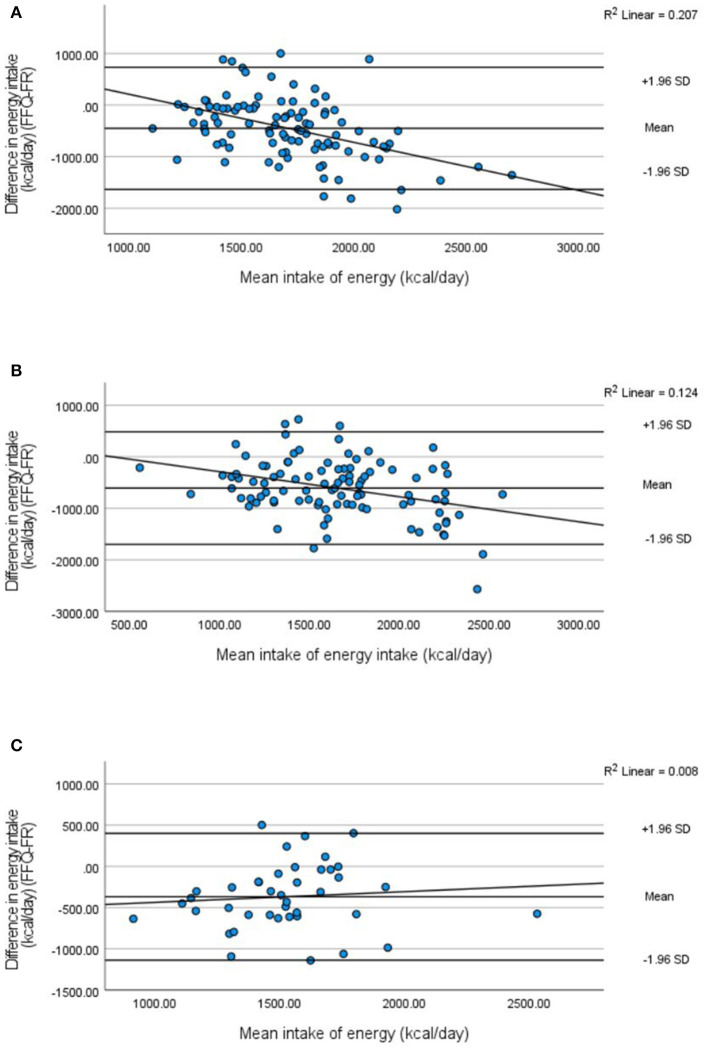
Bland–Altman plots with regression lines for energy intake measured by the FFQ compared with the 3-day food record in 2013 **(A)**, 2019 **(B)**, and 2021 **(C)**.

In general, the validation studies in 2013, 2019, and 2021 show that the FFQ underestimates energy and absolute macronutrient intake. Table 3 presents the convergent validity parameters for absolute macronutrients and macronutrient densities and Table 4 for micronutrients and micronutrient densities for the validation studies. A mean difference in energy intake shows an underestimation of the FFQ compared with the food record (FR) of 468.02 kcal/day (24.0%), 599.81 kcal/day (30.8%), and 350.87 kcal/day (20.6%) in 2013, 2019, and 2021, respectively (Table 3). As for energy intake, the mean difference for absolute protein, total fat, saturated fat, total carbohydrate, and fiber intake decreases over time (2013 vs. 2021) (Table 3). Spearman's correlations between the FFQ and FR for energy and absolute macronutrient intake ranged from 0.05 to 0.35 in 2013, from 0.16 to 0.44 in 2019, and from 0.02 to 0.54 in 2021, indicating moderate correlation (Table 3). Bland–Altman plots indicate good agreement between the FFQ and the FR for absolute macronutrient intake except for energy intake (Figures 2, 3). The Bland–Altman plots confirm an underestimation of the FFQ compared with the FR and show a downward regression trend in the data points indicating bias increases with increasing intake (Figure 3). The results of 2019 and 2021 show an overestimation of all macronutrient density intakes except for saturated fatty acid (SFA) intake (Table 3). Mean differences in density intakes are highest for MUFA and polysaccharide densities in 2013 and 2019 (10.13 and 20.41% in 2013 and 10.76 and 20.09% in 2019, respectively). Spearman's correlations between the FFQ and the FR for macronutrient density intake indicate moderate correlation and ranged from 0.02 to 0.47 in 2013, from 0.05 to 0.42 in 2019, and from 0.17 to 0.49 in 2021 (Table 3). Bland–Altman plots show good agreement for all macronutrient density intakes of the FFQ compared with the FR (figures not shown). Mean differences in absolute micronutrient intake between the FFQ and the FR show an underestimation of the FFQ compared with the FR (Table 4). Low-to-moderate correlations were found for absolute micronutrient intake with the highest correlations found for calcium intake (*r*^2^ = 0.44 *p* = 0.001, *r*^2^ = 0.53 *p* = 0.000, *r*^2^ = 0.53 *p* = 0.001 in 2013, 2019, and 2021, respectively) (Table 4).

**Table 3:** Parameters of convergent validity for absolute intake of macronutrients according to the FFQ and food record.

	**2013**, ***n*** = **100**				**2019**, ***n*** = **100**				**2021**, ***n*** = **42**			
	$\bar{x}$ _difference_^a^	*p* ^b^	**SCC** ^c^	*p*_SCC_	$\bar{x}$ _difference_^a^	*p* ^b^	**SCC** ^c^	**p**_SCC_	$\bar{x}$ _difference_^a^	*p* ^b^	**SCC** ^c^	*p*_SCC_
**Macronutrients absolute**												
Energy (kcal)	468.02	0.001	0.28	0.005	599.81	0.000	0.38	0.000	350.87	0.001	0.15	0.351
Protein (g)	21.74	0.001	0.24	0.019	10.99	0.000	0.44	0.000	3.02	0.256	0.41	0.007
Total fat (g)	27.56	0.001	0.33	0.001	17.07	0.000	0.29	0.003	8.57	0.024	0.14	0.367
SFA (g)	11.91	0.001	0.34	0.001	11.90	0.000	0.38	0.000	8.95	0.001	0.26	0.098
MUFA (g)	9.24	0.001	0.31	0.002	12.41	0.000	0.22	0.026	9.58	0.001	0.02	0.918
PUFA (g)	4.50	0.001	0.27	0.007	−0.34	0.479	0.16	0.120	−0.88	0.170	0.31	0.043
Total carbohydrates (g)	28.18	0.001	0.27	0.007	46.29	0.000	0.37	0.000	17.52	0.053	0.31	0.048
Mono-/disaccharides (g)	7.19	0.197	0.05	0.595	8.71	0.028	0.26	0.010	−13.37	0.002	0.52	0.001
Polysaccharides (g)	5.89	0.142	0.35	0.001	33.72	0.000	0.36	0.000	21.13	0.001	0.34	0.029
Fiber (g)	4.46	0.001	0.35	0.001	3.81	0.000	0.37	0.000	1.00	0.310	0.54	0.001
**Macronutrient densities**												
Protein (En%)	0.90	0.054	0.02	0.818	−3.99	0.000	0.25	0.013	−3.19	0.001	0.19	0.239
Total fat (En%)	4.53	0.001	0.46	0.001	−4.88	0.000	0.22	0.026	−4.15	0.001	0.36	0.019
SFA (En%)	2.40	0.001	0.47	0.001	1.58	0.000	0.23	0.025	2.02	0.001	0.43	0.004
MUFA (En%)	−10.13	0.001	0.29	0.003	−10.76	0.001	0.05	0.647	−10.22	0.001	0.17	0.291
PUFA (En%)	−6.27	0.001	0.20	0.045	−6.92	0.001	0.04	0.680	−2.07	0.001	0.19	0.241
Total carbohydrates (En%)	−5.46	0.001	0.37	0.001	−4.73	0.000	0.42	0.000	−6.15	0.001	0.49	0.001
Mono-/disaccharides (En%)	−4.14	0.001	0.23	0.025	−4.83	0.000	0.22	0.031	−8.39	0.001	0.33	0.034
Polysacharides (En%)	−20.41	0.001	0.19	0.058	−20.09	0.001	0.18	0.082	−0.26	0.001	0.38	0.015

${}^{a}{\bar{x}}_{difference}$: Mean difference = Mean intake Food Record – Mean intake FFQ.

^*b*^Wilcoxon signed-rank test.

^*c*^SCC: Spearman's correlation coefficient.

**Table 4:** Parameters of convergent validity for absolute intake of micronutrients according to the FFQ and food record.

	**2013**, ***n*** = **100**				**2019**, ***n*** = **100**				**2021**, ***n*** = **42**			
	$\bar{x}$ _difference_^a^	*p* ^b^	**SCC** ^c^	*p*_SCC_	$\bar{x}$ _difference_^a^	*p* ^b^	**SCC** ^c^	**p**_SCC_	$\bar{x}$ _difference_^a^	*p* ^b^	**SCC** ^c^	*p*_SCC_
**Micronutrients absolute**												
Na (mg)	1173.08	0	0.19	0.058	923.86	0.000	0.37	0.000	545.38	0	0.04	0.784
K (mg)	591.41	0	0.17	0.102	355.84	0.000	0.31	0.002	60.95	0.59	0.32	0.039
Ca (mg)	201.27	0	0.44	0.001	230.48	0.000	0.53	0.000	110.45	0	0.53	0.001
P (mg)	312.11	0	0.31	0.002	384.09	0.000	0.45	0.000	204.41	0	0.50	0.001
Fe (mg)	2.91	0	0.19	0.057	0.63	0.045	0.19	0.065	−0.01	0.98	0.45	0.003
Vitamin B12 (μg)	1.66	0	0.10	0.343	−0.26	0.039	0.27	0.007	−0.84	0	0.32	0.038
Vitamin C (mg)	40.00	0	0.34	0.001	27.08	0.000	0.18	0.079	31.08	0	0.10	0.542
Vitamin D (μg)	3.86	0	0.24	0.015	9.33	0.006	0.15	0.137	6.55	0.04	0.16	0.304
**Micronutrient densities**												
Na (mg/100 kcal)	37.98	0	0.05	0.617	96.96	0.002	0.19	0.063	5.27	0.24	−0.06	0.702
K (mg/100 kcal)	−6.84	0	0.43	0.001	42.28	0.001	0.40	0.000	−35.87	0	−0.05	0.778
Ca (mg/100 kcal)	1.64	0	0.60	0.001	74.75	0.261	0.53	0.000	−34.14	0.05	0.47	0.002
P (mg/100 kcal)	1.42	0	0.25	0.012	43.69	0.025	0.44	0.000	69.60	0.03	0.49	0.001
Fe (mg/100 kcal)	0.02	0	0.03	0.799	0.36	0.001	0.27	0.007	−43.38	0	0.14	0.388
Vitamin B12 (mg/100 kcal)	0.03	0	0.17	0.083	0.44	0.001	0.38	0.000	−0.11	0	0.27	0.097
Vitamin C (mg/100 kcal)	1.31	0	0.45	0.001	7.58	0.663	0.18	0.077	0.55	0.52	0.01	0.970
Vitamin D (μg/100 kcal)	0.15	0	0.23	0.023	5.68	0.878	0.22	0.027	0.31	0.98	0.15	0.335

${}^{a}{\bar{x}}_{difference}$: Mean difference = Mean intake Food Record – Mean intake FFQ.

^*b*^Wilcoxon signed-rank test.

^*c*^SCC: Spearman's correlation coefficient.

#### 3.3.2. Food groups

**Figure 4:**
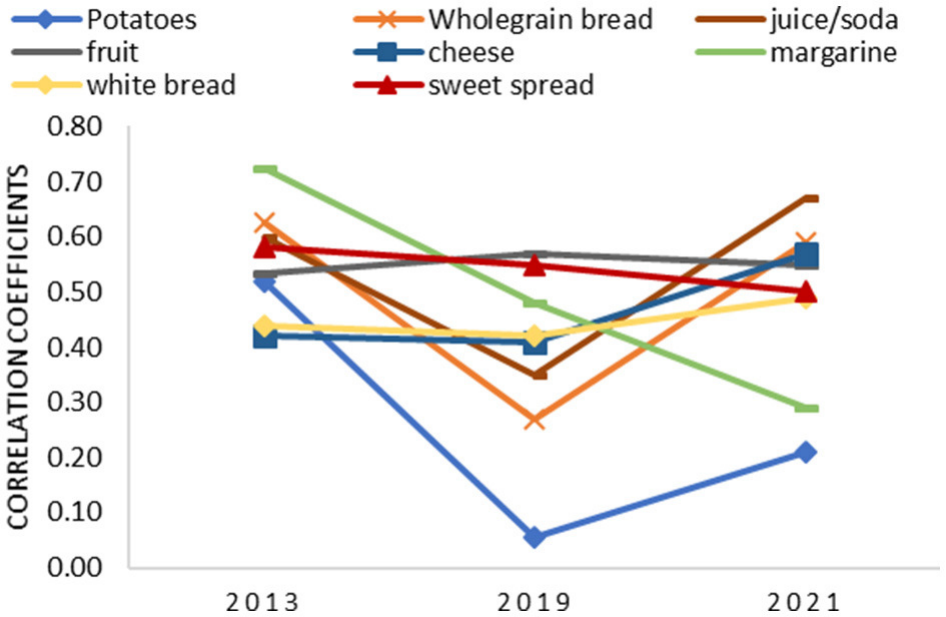
Correlation coefficients of different food groups in 2013, 2019, and 2021.

The results of the validity analysis for food group intake are presented in Table 5. Mostly moderate-to-good correlations were found. The highest correlation was found for alcoholic beverages (*r*^2^ = 0.81 *p* < 0.001) in 2013, coffee (*r*^2^ = 0.84 *p* = < 0.001) in 2019, and juices and soda (*r*^2^ = 0.67 *p* = 0.001) in 2021. The FFQ both underestimates and overestimates the intake of certain food groups. The results show that meat intake was underestimated by the FFQ by 19.42 g/day in 2013, by 25.98 g/day in 2019, and by 6.20 g/day in 2021. Full-fat dairy product intake, in contrast, was overestimated by the FFQ by 1.29 g/day in 2013, by 18.20 g/day in 2019, and by 18.46 g/day in 2021. As represented in Figure 4, correlations for intake of some food groups were similar across the three validation studies in 2013, 2019, and 2021 which was the case for the food groups :“fruit,” “cheese,” “white bread,” and “sweet spreads.” For the food group “margarine,” a decreasing correlation was found across the 3 years. Correlations of other food groups are lower in the validation study of 2019 compared with 2013 and 2021, such as for “potatoes,” “wholegrain bread,” and “juices and soda” (Figure 4). The results for convergent validity analysis after the exclusion of non-consumers are shown in Table 5. Consumers-only analysis shows lower correlations for most food groups measured by the FFQ compared with the food record (Supplementary Table S3). Consumers-only analysis shows a decrease in mean difference for most food groups compared analyses including all participants. The food groups “vegetarian meat substitute” and “wholegrain pasta and rice” had the lowest number of consumers, while the food groups “vegetables,” “meat,” and “sweet snacks” had the highest number of consumers across the 3 years.

**Table 5:** Convergent validity of food group intake according to the FFQ and the food record.

	**2013**, ***n*** = **100**				**2019**, ***n*** = **100**				**2021**, ***n*** = **42**			
	$\bar{x}$ _difference_^a^	*p* ^b^	**SCC** ^c^	*p*_SCC_	$\bar{x}$ _difference_^a^	*p* ^b^	**SCC** ^c^	**p**_SCC_	$\bar{x}$ _difference_^a^	*p* ^b^	**SCC** ^c^	*p*_SCC_
**Food groups**												
Potatoes	2.65	0.678	0.52	0.000	−11.05	0.051	0.06	0.577	−2.93	0.712	0.21	0.187
Alcoholic beverages	60.26	0.000	0.81	0.000	54.84	0.611	0.39	0.000	−3.64	0.662	0.54	0.001
Cooking fat/oil	−5.95	0.000	0.25	0.014	−10.64	0.000	−0.01	0.920	−11.22	0.001	0.04	0.826
Wholegrain bread	3.04	0.497	0.63	0.000	−6.28	0.144	0.27	0.008	−27.71	0.001	0.59	0.001
Wholegrain pasta/rice	−11.11	0.000	0.26	0.009	−26.09	0.000	0.09	0.399	−29.16	0.001	0.30	0.056
Cornflakes/cruesli					4.13	0.197	0.47	0.000	6.45	0.062	0.46	0.002
Eggs	5.83	0.000	0.28	0.005	−0.05	0.583	0.28	0.005	−2.56	0.418	0.39	0.012
Juice/soda	−22.61	0.404	0.60	0.000	51.43	0.040	0.35	0.000	−36.98	0.121	0.67	0.001
Fruit	−26.23	0.022	0.53	0.000	−10.35	0.310	0.57	0.000	−68.86	0.001	0.55	0.001
Vegetables	−18.54	0.064	0.28	0.005	−0.44	0.975	0.29	0.004	−9.22	0.664	0.05	0.748
Cheese	13.97	0.000	0.42	0.000	22.49	0.000	0.41	0.000	17.45	0.001	0.57	0.001
Coffee					−53.09	0.000	0.84	0.000	−83.90	0.033	0.64	0.001
Skimmed dairy products	−72.82	0.000	0.51	0.000	−56.86	0.000	−0.03	0.791	−63.95	0.001	0.39	0.320
Oats/wholegrain cereals	1.16	0.327	0.69	0.000	−5.28	0.001	0.58	0.000	−8.18	0.055	0.56	0.001
Nuts/seeds					−2.53	0.000	0.45	0.000	−7.46	0.002	0.19	0.239
Legumes					0.65	0.263	0.19	0.061	1.43	0.618	0.15	0.340
Sauces	22.73	0.000	0.20	0.045	27.04	0.000	0.12	0.250	17.53	0.001	0.20	0.217
Margarine	3.16	0.040	0.72	0.000	−1.87	0.007	0.48	0.000	−1.15	0.293	0.29	0.062
Sugared dairy products					−4.91	0.075	0.20	0.051	−8.94	0.251	0.12	0.442
Soup					−15.24	0.002	0.32	0.001	−6.41	0.758	0.26	0.099
Fish	3.68	0.329	0.24	0.017	1.76	0.156	0.30	0.002	5.89	0.185	0.37	0.015
Fish spread	−1.04	0.434	0.22	0.033	−2.28	0.000	0.13	0.211	−1.25	0.539	0.18	0.254
Vegetarian meat substitute	3.83	0.069	0.56	0.000	0.95	0.260	0.30	0.002	0.06	0.977	0.27	0.090
Meat	19.42	0.002	0.34	0.001	25.98	0.004	0.44	0.000	6.20	0.356	0.44	0.003
Deli meats	12.01	0.000	0.38	0.000	11.55	0.021	0.40	0.000	1.58	0.648	0.41	0.006
Full fat dairy products	−1.29	0.907	0.11	0.261	−18.20	0.022	0.14	0.177	−18.46	0.087	0.55	0.001
Water/tea	21.99	0.649	0.51	0.000	−769.30	0.000	0.03	0.750	−954.38	0.001	−0.17	0.285
White bread	15.07	0.000	0.44	0.000	34.07	0.000	0.42	0.000	15.93	0.007	0.49	0.001
White pasta/rice	7.78	0.182	0.09	0.385	12.57	0.085	0.22	0.032	40.64	0.001	0.22	0.172
Sweet spread	4.71	0.004	0.58	0.000	2.11	0.230	0.55	0.000	1.69	0.437	0.50	0.001
Sweet snacks	25.88	0.000	0.29	0.004	23.00	0.000	0.25	0.014	31.31	0.001	0.14	0.376
Savory snacks					−5.46	0.000	0.27	0.007	−5.19	0.024	0.42	0.005

${}^{a}{\bar{x}}_{difference}$: Mean difference = Mean intake Food Record – Mean intake FFQ.

^*b*^Wilcoxon signed-rank test.

^*c*^SCC: Spearman's correlation coefficient.

### 3.4. Ranking and misclassification

Cross-classification analysis by quartiles is presented in Tables 6, 7. Correct classification of absolute macronutrient intake by the FFQ into the same or adjacent quartile ranged from 60 to 78% in 2013, from 68 to 80% in 2019, and from 68.7 to 83.3% in 2021 (Table 6). Misclassification of absolute macronutrient intake was < 10%, except for monosaccharides and disaccharides (11.0%) in 2013, for PUFA (10.0%) in 2019, and for energy (11.9 %) in 2021. The validation study of 2013 showed acceptable agreement (κ ≥ 0.20) for absolute intake of total fat, SFA, MUFA, polysaccharides, and fiber. In 2019, an acceptable agreement was found for the absolute intake of protein and fiber, while in 2021, absolute intake of PUFA and all carbohydrates showed acceptable agreement between the FFQ and the food record (Table 6). Improvement in misclassification was seen between 2013 and 2021 for all macronutrient density intakes, except for SFA, MUFA, and PUFA densities. Agreement between ranking participants according to their intake between the methods increases for macronutrient densities compared with absolute macronutrient intake across the 3 years for total fat, SFA, and total carbohydrate. The highest weighted kappa coefficients for micronutrient intake were found for calcium in 2013 (κ = 0.24, *p* = 0.001) and 2019 (κ = 0.18, *p* = 0.000) and for calcium, phosphorus, and iron in 2021 (κ = 0.34, *p* = 0.002) for all three micronutrients (Table 7).

**Table 6:** Cross-classification analysis of Food Frequency Questionnaire compared with a 3-day food record for macronutrients.

	**2013**, ***n*** = **100**				**2019**, ***n*** = **100**				**2021**, ***n*** = **42**			
	**Same or adjacent quartile**	**Opposite quartile**	**Weighted kappa**	***p***	**Same or adjacent quartile**	**Opposite quartile**	**Weighted kappa**	***p***	**Same or adjacent quartile**	**Opposite quartile**	**Weighted kappa**	***p***
**Macronutrients absolute**												
Energy (kcal)	61.0%	7.0%	0.18	0.011	80.0%	6.0%	0.09	0.018	68.7%	11.9%	0.11	0.334
Protein (g)	71.0%	7.0%	0.13	0.075	80.0%	4.0%	0.26	0.000	78.6%	4.8%	0.18	0.095
Total fat (g)	74.0%	7.0%	0.24	0.001	74.0%	7.0%	0.10	0.082	71.4%	9.4%	0.07	0.540
SFA (g)	78.0%	7.0%	0.25	0.001	79.0%	5.0%	0.05	0.149	73.8%	7.2%	0.11	0.334
MUFA (g)	75.0%	5.0%	0.22	0.002	70.0%	7.0%	0.07	0.059	66.7%	2.4%	0.03	0.793
PUFA (g)	74.0%	7.0%	0.15	0.035	68.0%	10.0%	0.09	0.172	76.1%	4.8%	0.26	0.017
Total carbohydrates (g)	70.0%	6.0%	0.18	0.012	78.0%	6.0%	0.15	0.003	76.1%	7.2%	0.23	0.037
Mono-/disaccharides (g)	60.0%	11.0%	0.03	0.655	73.0%	9.0%	0.16	0.009	83.3%	2.4%	0.34	0.002
Polysaccharides (g)	73.0%	6.0%	0.23	0.003	79.0%	8.0%	0.15	0.002	81.0%	7.2%	0.22	0.043
Fiber (g)	77.0%	7.0%	0.22	0.002	76.0%	5.0%	0.20	0.001	80.9%	2.4%	0.38	0.001
**Macronutrient densities**												
Protein (En%)	61.0%	10.0%	−0.01	0.911	71.0%	6.0%	0.12	0.001	66.6%	7.2%	0.07	0.540
Total fat (En%)	78.0%	3.0%	0.26	0.001	74.0%	9.0%	0.13	0.012	71.4%	9.5%	0.18	0.095
SFA (En%)	79.0%	5.0%	0.29	0.001	68.0%	8.0%	0.10	0.075	81.0%	2.4%	0.26	0.017
MUFA (En%)	68.0%	4.0%	0.12	0.096	63.0%	13.0%	0.03	0.657	64.3%	4.8%	0.07	0.549
PUFA (En%)	65.0%	5.0%	0.09	0.205	63.0%	9.0%	0.04	0.574	61.9%	7.2%	0.11	0.297
Total carbohydrates (En%)	74.0%	5.0%	0.24	0.001	84.0%	6.0%	0.22	0.000	83.3%	2.4%	0.34	0.002
Mono-/Disaccharides (En%)	75.0%	11.0%	0.20	0.007	68.0%	9.0%	0.11	0.021	76.2%	7.2%	0.30	0.006
Polysaccharides (En%)	66.0%	7.0%	0.10	0.149	69.0%	9.0%	0.12	0.096	76.2%	0.0%	0.226	0.037

**Table 7:** Cross-classification analysis of Food Frequency Questionnaire compared with a 3-day food record for micronutrients.

	**2013**, ***n*** = **100**				**2019**, ***n*** = **100**				**2021**, ***n*** = **42**			
	**Same or adjacent quartile**	**Opposite quartile**	**Weighted kappa**	***p***	**Same or adjacent quartile**	**Opposite quartile**	**Weighted kappa**	***p***	**Same or adjacent quartile**	**Opposite quartile**	**Weighted kappa**	***p***
**Micronutrients absolute**												
Na (mg)	56.0%	24.0%	0.08	0.180	81.0%	4.0%	0.09	0.007	64.3%	9.5%	−0.01	0.926
K (mg)	64.0%	8.0%	0.05	0.506	75.0%	8.0%	0.17	0.004	69.0%	4.8%	0.18	0.095
Ca (mg)	75.0%	3.0%	0.24	0.001	82.0%	4.0%	0.18	0.000	88.1%	4.8%	0.34	0.002
P (mg)	69.0%	7.0%	0.14	0.059	80.0%	3.0%	0.14	0.001	83.4%	0.0%	0.34	0.002
Fe (mg)	61.0%	6.0%	0.06	0.375	69.0%	9.0%	0.10	0.105	81.0%	4.8%	0.34	0.002
Vitamin B12 (μg)	68.0%	10.0%	0.10	0.178	73.0%	8.0%	0.13	0.023	71.5%	4.8%	0.17	0.118
Vitamin C (mg)	54.0%	11.0%	0.12	0.036	65.0%	12.0%	0.05	0.363	69.1%	9.5%	0.11	0.334
Vitamin D (μg)	73.0%	6.0%	0.15	0.043	65.0%	10.0%	0.08	0.135	73.8%	9.6%	0.15	0.187
**Micronutrient densities**												
Na (mg/100 kcal)	63.0%	12.0%	−0.01	0.912	69.0%	12.0%	0.08	0.247	52.3%	9.6%	−0.08	0.487
K (mg/100 kcal)	70.0%	10.0%	0.17	0.007	77.0%	7.0%	0.27	0.001	59.4%	14.3%	−0.09	0.385
Ca (mg/ 100 kcal)	82.0%	0.0%	0.41	0.001	80.0%	3.0%	0.33	0.001	81.0%	7.2%	0.28	0.009
P (mg/100 kcal)	63.0%	8.0%	0.18	0.003	69.0%	7.0%	0.25	0.001	76.2%	9.6%	0.18	0.053
Fe (mg/100 kcal)	66.0%	7.0%	0.08	0.276	72.0%	13.0%	0.13	0.071	66.7%	11.9%	0.11	0.301
Vitamin B12 (μg/100 kcal)	73.0%	10.0%	0.15	0.044	73.0%	3.0%	0.22	0.002	71.4%	7.2%	0.03	0.814
Vitamin C (mg/100 kcal)	79.0%	3.0%	0.27	0.001	68.0%	8.0%	0.09	0.205	66.2%	14.3%	0.11	0.297
Vitamin D (μg/100 kcal)	73.0%	8.0%	0.10	0.189	69.0%	9.0%	0.18	0.012	61.8%	9.6%	0.04	0.729

### 3.5. Reliability

The reliability of the FFQ was assessed in 2013 and 2019 by test–retest assessment of 84 participants in 2013 and 81 participants in 2019 (Tables 8, 9). Wilcoxon signed-rank tests show that there was no statistically significant difference between the two administrations of the FFQ in 2013 for macronutrient intake, macronutrient densities, and micronutrient intake, except for energy intake. Spearman's correlation coefficients between the two administrations show good reliability and range from 0.51 to 0.75 (*p* < 0.005) in 2013 and from 0.58 to 0.81 (*p* < 0.005) in 2019 (Table 7). Intra-class correlation coefficients show a similar trend. Both Spearman's correlation coefficients and intra-class correlations improve further between 2013 and 2019 for energy intake and intake of most absolute macronutrients. However, for all macronutrient density intakes, the correlation coefficients between the two administrations of the FFQ decreases in 2019 compared with 2013, except for protein, MUFA, and PUFA density (Table 7).

**Table 8:** Reliability of the Food Frequency Questionnaire assessed by test–retest in 2013 and 2019 for macronutrients.

	***n*** = **84**						***n*** = **81**					
	**Mean (SE)**_FFQ1_	**Mean (SE)**_FFQ2_	*p* ^a^	**SCC** ^b^	**ICC** ^c^	**95% CI**	**Mean (SE)**_FFQ1_	**Mean (SE)**_FFQ2_	*p* ^a^	**SCC** ^b^	**ICC** ^c^	**95% CI**
**Macronutrients absolute**												
Energy (kcal)	1480.0 (34.0)	1432.7 (36.4)	0.04	0.66^*^	0.68	0.548; 0.781	1347.0 (45.7)	1292.9 (42.2)	0.08	0.72^*^	0.74	0.625; 0.826
Protein (g)	59.7 (1.6)	58.4 (1.5)	0.51	0.53^*^	0.53	0.354; 0.666	71.0 (2.5)	67.2 (2.3)	0.02	0.72^*^	0.72	0.589; 0.808
Total fat (g)	51.9 (1.4)	50.1 (1.5)	0.10	0.64^*^	0.66	0.524; 0767	60.1 (2.4)	56.7 (2.3)	0.04	0.70^*^	0.73	0.607; 0.817
SFA (g)	19.5 (0.5)	18.9 (0.6)	0.08	0.67^*^	0.66	0.519; 0.764	17.7 (0.7)	17.2 (0.7)	0.22	0.81^*^	0.83	0.746; 0.886
MUFA (g)	18.9 (0.6)	18.4 (0.6)	0.34	0.63^*^	0.64	0.497; 0.752	18.6 (7.0)	17.8 (0.8)	0.10	0.81^*^	0.84	0.760; 0.894
PUFA (g)	11.6 (0.4)	11.1 (0.4)	0.07	0.62^*^	0.66	0.521; 0.766	11.3 (0.5)	10.7 (0.5)	0.05	0.76^*^	0.80	0.701; 0.866
Total carbohydrates (g)	179.5 (5.3)	174.0 (5.4)	0.08	0.64^*^	0.71	0.580; 0.798	158.8 (6.1)	150.6 (5.5)	0.05	0.66^*^	0.69	0.552; 0.787
Mono-/disaccharides (g)	82.7 (3.2)	81.1 (2.8)	0.21	0.70^*^	0.73	0.613; 0.817	64.3 (2.3)	65.0 (2.3)	0.48	0.71^*^	0.66	0.521; 0.770
Polysaccharides (g)	96.5 (3.3)	92.6 (3.8)	0.12	0.67^*^	0.67	0.534; 0.773	92.7 (4.4)	85.5 (3.7)	0.04	0.64^*^	0.67	0.524; 0.773
Fiber (g)	17.5 (0.6)	17.2 (0.6)	0.77	0.68^*^	0.70	0.569; 0.793	15.2 (0.7)	14.3 (0.6)	0.03	0.71^*^	0.70	0.568; 0.795
**Macronutrient densities**												
Protein (En%)	16.2 (0.3)	16.4 (0.3)	0.22	0.61^*^	0.67	0.538; 0.775	21.2 (0.4)	20.9 (0.4)	0.32	0.63^*^	0.65	0.499; 0.757
Total fat (En%)	31.6 (0.6)	31.6 (0.6)	0.90	0.70^*^	0.7	0.572; 0.795	40.2 (0.7)	39.1 (0.8)	0.16	0.58^*^	0.62	0.461; 0.734
SFA (En%)	11.9 (0.2)	11.9 (0.2)	0.86	0.75^*^	0.74	0.624; 0.823	11.8 (0.3)	12.0 (0.3)	0.65	0.69^*^	0.69	0.554; 0.788
MUFA (En%)	11.5 (0.3)	11.6 (0.2)	0.10	0.63^*^	0.64	0.497; 0.752	12.4 (0.3)	12.3 (0.3)	0.514	0.67^*^	0.70	0.573; 0.797
PUFA (En%)	7.1 (0.2)	7.0 (0.2)	−0.08	0.62^*^	0.66	0.521; 0.766	7.5 (0.2)	7.3 (0.2)	0.215	0.67^*^	0.75	0.64; 0.83
Total carbohydrates (En%)	48.4 (7.0)	48.4 (6.2)	−0.08	0.70^*^	0.77	0.659; 0.841	46.9 (0.8)	46.6 (7.1)	0.4	0.68^*^	0.71	0.578; 0.801
Mono-/Disaccharides (En%)	22.4 (6.8)	22.9 (6.2)	−0.08	0.75^*^	0.76	0.649; 0.835	19.8 (0.6)	20.8 (5.8)	0.02	0.71^*^	0.74	0.614; 0.822
Polysaccharides (En%)	25.8 (0.5)	25.4 (0.6)	−0.49	0.67^*^	0.67	0.534; 0.773	26.8 (0.8)	26.1 (0.6)	0.304	0.63^*^	0.66	0.521; 0.769

^*a*^Wilcoxon signed-rank test.

^*b*^SCC, Spearman's correlation coefficient.

^*c*^ICC, Intra-class correlation coefficient.

^*^p < 0.05.

**Table 9:** Reliability of the Food Frequency Questionnaire assessed by test–retest in 2013 and 2019 for micronutrients.

	***n*** = **84**						***n*** = **81**					
	**Mean (SE)**_FFQ1_	**Mean (SE)**_FFQ2_	*p* ^a^	**SCC** ^b^	**ICC** ^c^	**95% CI**	**Mean (SE)**_FFQ1_	**Mean (SE)**_FFQ2_	*p* ^a^	**SCC** ^b^	**ICC** ^c^	**95% CI**
**Micronutrients absolute**												
Na (mg)	1519.6 (43.73)	1463.5 (49.6)	0.22	0.64^*^	0.66	0.516; 0.763	1589.9 (560.6)	1485.8 (479.9)	0.02	0.66^*^	0.67	0.527; 0.775
K (mg)	2615.4 (66.3)	2561.5 (64.6)	0.51	0.65^*^	0.64	0.491; 0.748	2383.9 (634.7)	2316.1 (601.5)	0.15	0.65^*^	0.67	0.531; 0.774
Ca (mg)	586.3 (22.06)	573.1 (21.5)	0.66	0.61^*^	0.63	0.482; 0.744	534.0 (206.3)	537.4 (203.8)	0.89	0.71^*^	0.69	0.552; 0.787
P (mg)	970.4 (24.2)	947.0 (23.4)	0.33	0.57^*^	0.55	0.385; 0.685	1012.4 (322.0)	969.9 (293.7)	0.05	0.71^*^	0.75	0.633; 0.830
Fe (mg)	9.1 (0.2)	8.9 (0.2)	0.66	0.62^*^	0.61	0.456; 0.728	9.9 (2.8)	9.3 (2.3)	0.01	0.65^*^	0.67	0.518; 0.774
Vitamin B12 (μg)	3.6 (0.1)	3.5 (0.1)	0.65	0.51^*^	0.54	0.370; 0.676	4.2 (1.63)	4.0 (1.6)	0.13	0.75^*^	0.75	0.642; 0.835
Vitamin C (mg)	66.8 (2.4)	65.1 (2.3)	0.39	0.67^*^	0.68	0.548; 0.781	48.6 (17.7)	47.6 (17.1)	0.39	0.69^*^	0.67	0.525; 0.772
Vitamin D (μg)	3.7 (0.1)	3.6 (0.1)	0.36	0.56^*^	0.50	0.323; 0.645	6.4 (3.6)	5.8 (2.9)	0.09	0.61^*^	0.69	0.549; 0.788
**Micronutrient densities**												
Na (mg/100 kcal)	102.9 (1.7)	101.7 (1.9)	0.80	0.62^*^	0.60	0.442; 0.720	119.0 (3.1)	115.9 (2.7)	0.299	0.49^*^	0.57	0.401; 0.698
K (mg/100 kcal)	177.8 (3.2	180.8 (3.2)	0.21	0.73^*^	0.80	0.706; 0.864	182.9 (4.0)	184.0 (3.5)	0.626	0.68^*^	0.68	0.544; 0.783
Ca (mg/100 kcal)	40.1 (1.4)	40.8 (1.5)	0.26	0.67^*^	0.73	0.615; 0.818	40.8 (1.4)	42.4 (1.4)	0.324	0.64^*^	0.63	0.476; 0.743
P (mg/100 kcal)	65.9 (1.03)	66.8 (1.0)	0.30	0.67^*^	0.73	0.607; 0.813	75.9 (1.3)	75.9 (1.2)	0.206	0.68^*^	0.67	0.531; 0.774
Fe (mg/100 kcal)	0.6 (0.0)	0.6 (0.0)	0.18	0.71^*^	0.74	0.620; 0.821	0.8 (0.0)	0.7 (1.2)	0.994	0.67^*^	0.69	0.555; 0.789
Vitamin B12 (μg/100 kcal)	0.3 (0.0)	0.3 (0.0)	0.15	0.65^*^	0.71	0.588; 0.803	0.3 (0.0)	0.3 (0.0)	0.761	0.70^*^	0.72	0.596; 0.810
Vitamin C (mg/100 kcal)	4.5 (0.1)	4.6 (0.1)	0.48	0.72^*^	0.74	0.623; 0.822	3.8 (0.2)	3.8 (0.1)	0.783	0.73^*^	0.74	0.625; 0.826
Vitamin D (μg/100 kcal)	0.3 (0.0)	0.3 (0.0)	0.99	0.64^*^	0.64	0.494; 0.751	0.5 (0.0)	0.5 (0.0)	0.211	0.56^*^	0.58	0.413; 0.705

^*a*^Wilcoxon signed-rank test.

^*b*^SCC, Spearman's correlation coefficient.

^*c*^ICC, Intra-class correlation coefficient.

^*^p < 0.05.

## 4. Discussion

### 4.1. Summary

In general, the FFQ underestimates energy intake and absolute macro- and micronutrient intake but shows good reliability and acceptable agreement compared with the food record. The estimation of macronutrient density by the FFQ resulted in a small overestimation compared with the food record. However, the overestimation is < 5% for most nutrient densities and is therefore considered acceptable. Low-to-moderate correlations were found for convergent validity of the FFQ compared with the food record for both macro- and micronutrients. However, the Bland–Altman plots provide better insight and showed good agreement between the FFQ and the food record, indicating acceptable accuracy of the FFQ taking the systematic bias into account. Interestingly, the Bland–Altman plots show a clear improvement in the FFQ over the years. In the first validation study in 2013, a strong downward regression trend was present revealing that underestimation increases with higher intake. In 2019, the FFQ was validated for its content and adapted accordingly, next to adding more specific food groups based on the new Flemish Food-Based Dietary Guidelines published in 2019. This could have contributed to the decreased downward regression between intake and underestimation seen on the 2019 Bland–Altman plots until the plot of data in 2021 shows a complete disappearance of downward regression trend and smaller limits of agreement. However, the cohort of 2021 had a considerably smaller sample size (*n* = 42) compared with the cohorts of 2013 and 2019 (*n* =1 00 and *n* =1 00, respectively), and therefore, the regression trend should be interpreted with caution. For assessing food group intake, the FFQ shows acceptable to good convergent validity but differs greatly between the food groups. Consumers-only analysis results in smaller correlation coefficients compared with validity analysis for all consumers. However, FFQs are not developed to assess absolute nutrient or food group intake but are rather used for ranking populations according to their intake, and misclassification is an important aspect to assess. Generally, misclassification of 10% or less is preferred, which has been achieved by the FFQ for most nutrients and food groups. However, Cohen's kappa values were generally low, which could be caused by chance agreement. Finally, the reliability of the FFQ was shown to be good indicating great precision of the FFQ.

### 4.2. Comparison with the literature

The results of the validation studies of our FFQ are in line with other similar validation studies of FFQs. However, comparison of validity results requires caution as these studies differ greatly depending on the questionnaire length, reference method used, number of administrations of the reference method, and study population, for example (6, 25). Moreover, validity is not always assessed together with reproducibility, cross-classification, or misclassification analysis. While some studies show that FFQs overestimate measurements, others show an underestimation, similar to our study (26–28). A meta-analysis by Cui et al. examined the validity of FFQs compared with both 24-h recalls and food records and concluded that the number of administrations of the reference method, gender, sample size, and reference period impacts the validity correlation (6). Steineman et al. assessed the convergent validity of a paper-based FFQ compared with a 4-day food record (26). The FFQ of Steineman et al. consisted of 127 items assessing the intake of 25 food groups in the past 4 weeks. This FFQ was considerably more extensive compared with our FFQ and differed by the use of three standard portion size options in their FFQ (small, medium, and large) and the use of nine answer options to indicate the frequency of consumption (26). However, correlation coefficients were similar to our results for nutrients and food intake and ranged from 0.27 to 0.55 and from 0.09 to 0.92, respectively (26). Bland–Altman plots presented by Steineman et al. showed a similar trend as presented in our Bland–Altman plots and the study of Mumu et al., where bias increases with increasing intake for both men and women (29). Moreover, it was shown that energy-dense foods are underestimated more and women underestimate more than men (26). The study population by Mumu et al. consisted of ~60% women which is similar to our study samples of 2013 and 2019 (65 and 58%, respectively) (26). Women were represented in the same ratio in the study population of the FFQ validation study of Eysteinsdottir et al., in which a short FFQ with 30 questions was validated for food group intake against a 3-day weighed food record in people of advanced age (mean age = 74 years) (28). Similar correlation coefficients were found for food group intake between the FFQ and the weighed food record and ranged from 0.05 to 0.71 for men and from 0.01 to 0.61 for women. Although generally lower correlation coefficients were found compared with our study, the highest correlation coefficients were found for a beverage, specifically tea, as well. Remarkably, beverages including coffee, juice, and alcoholic beverages showed the highest correlation coefficients between the FFQ and the food record in our validation study as well. This was shown in other studies as well and may be explained that portion sizes of beverages (i.e., a glass, a mug, and a bottle) are easier to estimate compared with solid foods such as potatoes or meat, indicating that the ability to estimate portion size impacts validation results significantly (30). However, correlation coefficients for water intake were high at first in 2013 but very low in 2019 and 2021 (Table 3). This is possibly due to the different registration methods of the food record. In 2013, a paper-based food record, including probing questions toward specific food categories, was used while in 2019 and 2021, the MyFitnessPal application was used as a dietary record. Although MyFitnessPal can be used as a food record to calculate nutrient intake, the MyFitnessPal app in our validation study was solely used to capture the type of foods consumed while nutrient calculations were based on the Belgian Food Composition Table (NUBEL 2009) (20, 31). However, the use of MyFitnessPal to register the type of consumed foods only yielded some shortcomings. Water intake and also cooking fat use were mostly forgotten to be registered in the mobile application used as food record, leading to unrealistically low consumptions of water intake in the food record and low correlation coefficients between the FFQ and the food record for those food groups. Additionally, MyFitnessPal has a default option to register complete meals (i.e., Caesar salad or pasta dishes) which lead to larger error in estimated portions sizes compared to registering the individual foods of which the meal consisted.

Mumu et al. investigated the validity of a 126-item FFQ compared with 24-h recalls and biomarkers (29). While correlation coefficients were higher for nutrient intake, misclassification was found to be 18% on average, which is significantly higher compared with our findings of 7.7, 7.8, and 5.9% misclassification on average in 2013, 2019, and 2021, respectively.

Cui et al. performed a meta-analysis on the reproducibility of Food Frequency Questionnaires and identified factors influencing reproducibility of Food Frequency Questionnaires (25). Pooled ICC of macronutrients ranged from 0.51 for starch to 0.78 for fiber, which are similar to our findings with ICCs in 2013 ranging from 0.53 for protein to 0.71 for monosaccharides and disaccharides. Remarkably, fiber was also the nutrient to have amongst the highest ICC (ICC = 0.70) in our study of 2013, and starch was the nutrient with lowest ICC of 0.64 in our study of 2019 as well.

### 4.3. Factors influencing the validity

However, most validation studies use 24-h recalls as a reference method and therefore cannot be compared directly. As 24-h recalls and FFQs are known to have correlated errors, this can explain higher correlation coefficients found in validation studies, using 24-h recalls as the reference method for the FFQ compared with food records. The meta-analysis of Cui et al. showed that correlation coefficients in validation studies using 24-h recalls are higher compared with studies using food records as a reference method. Moreover, Molag et al. showed that correlation coefficients increased when the reference method was used for longer periods, specifically 8 to 14 days (32). The food records in our validation studies consisted of 3-day records only. However, this choice is based on research showing that the quality and completeness of the food records decrease significantly after 4 days while the burden increases to register dietary intake for more days (1).

Another factor influencing the validity of the FFQ is the length of the questionnaire. Most validation studies include FFQs with more than 100 food items, while our FFQ is considerably shorter consisting of 33 food items. Molag et al. confirm that FFQs with more than 100 food items found generally higher correlation coefficients and perform better at ranking subjects, according to their intake (32). Block et al. highlight that reproducibility is higher too for shorter questionnaires (33). Moreover, the use of portion size questions (quantitative vs. qualitative) in FFQs seems to result in higher correlation coefficients as well compared with the use of predefined portions (32). Finally, categorization of mixed dishes in the food record into different food groups influences obtained energy and macronutrient intakes and may influence the outcome of convergent validity accordingly (34).

Marks et al. investigated how gender, age, and BMI influenced the convergent validity of the FFQ compared with a 12-day weighed food record (30). The bias of the FFQ compared with the food record was different for men and women for different foods. Moreover, the study by Marks et al. showed that age was associated with differences between the FFQ and the food record for specific foods including vegetables and cereals (product). Additionally, BMI was shown to be not significantly associated with such differences. However, difference between intakes measured by the FFQ compared with the food record increases with increasing BMI or age, which is a possible explanation for the higher difference between the FFQ and the food record seen in the Bland–Altman plots.

### 4.4. Changing validity over time

It is difficult to assess whether validation has improved over the years as the differences in sample size, study population characteristics (i.e., age), update of the FFQ, different registration method food record (paper-based FR vs. MyFitnessPal), and the changing dietary pattern during the past decades could explain different outcomes in validation as well. Although Spearman's correlation coefficients seem not to improve significantly for macronutrient intake, Bland–Altman plots do show less bias with increasing intake and smaller limits of agreement, indicating improved accuracy. On the contrary, convergent validity of the FFQ seemed to be improved for micronutrient intake, except for sodium, vitamin C, and vitamin D. Convergent validity of food group intake has decreased over the years, showing lower correlation coefficients and intake of most food groups being statistically differently measured by the FFQ compared with the food record. However, these results are probably influenced greatly by the changing dietary pattern and the different age groups, having different and more irregular dietary patterns with more inter-person and between-person variability in the young study population of 2019. Moreover, it can be argued that the food groups questioned in the FFQ are better resembling the “traditional” dietary pattern of the older study population in 2013 and less suitable for study populations of 2019 and 2021 having different dietary patterns and habits including more diverse and “new” foods. The ranking capability of the FFQ seemed similar across the years with good misclassification rates but fair- to low-weighed kappa values. Additionally, comparison between the different cohorts is difficult due to differences in sample size as misclassification in smaller sample sizes can cause large differences in kappa values (35). Reproducibility, on the contrary, seemed to be improved between 2013 and 2019, showing good reproducibility of the FFQ. However, the period between the two administrations is rather short (1 and 3 weeks, respectively) and can have contributed to higher Spearman's correlations and ICCs.

### 4.5. Strengths and limitations

The strength of this research is the ability to assess the validation in different sample populations while both convergent validity, reliability together with consumers-only analysis, and cross-classification have been evaluated extensively in different time periods. As diet is culture-specific, diet changes also over time. In total, 10 years ago, potatoes and bread were food groups consumed at almost every meal in a typical Belgian diet. However, lately, more “new” foods have been introduced into the Belgian dietary pattern and gained popularity. This was reflected in the dietary records, as well, with food records of 2013 including mostly “traditional” meals with potatoes or bread as the major food group while the latest food records showed a shifted dietary pattern including considerably less bread but more “on-trend” foods, such as oatmeal, avocado, quinoa, and chia seeds. This trend can also be reinforced by the difference in age of the cohorts in the three different validation studies of 2013, 2019, and 2021. In 2013, the cohort was older while the sample population of 2019 consisted mainly of young students and the age of the sample population of 2021 was more generally representative but had a small sample size and was overall highly educated (*n* = 42). The food records of the students showed greater irregularities in the dietary pattern including skipping meals (i.e., breakfast), higher intake of alcoholic beverages, fast food, sweet snacks, and ready-to-eat meals. This is also represented as this study population has the largest mean difference for energy intake between the FFQ and the food record, which is mainly contributed to the discrepancies for mono- and disaccharides. Nevertheless, the data of the different cohorts should be compared with caution. The cohort of 2019 and 2021 used an updated version of the FFQ including 32 food items compared with the original FFQ used in 2013 with 27 items.

Additionally, a different method as food record was used in 2013 compared with 2019 and 2021. While a paper-based food record was used in 2013, the studies of 2019 and 2021 have used the MyFitnessPal application as a food record. The use of MyFitnessPal introduced serious pitfalls mainly because the application is developed originally for the United States (U.S.) while our study participants are Belgian. Although the MyFitnessPal application is used in Dutch, it is still embedded with US culture-specific features including US foods or recipes (i.e., Caesar Salad) and measuring units (i.e., cups). Moreover, study participants used US measuring units such as cups to indicate portion size in MyFitnessPal while participants are most likely unfamiliar with US units as metric units are the standard in Belgium. The main advantage of MyFitnessPal is being feasible and easy-to-use for participants, while the disadvantage includes the appeal to register meals as a whole (i.e., “one Caesar salad” for lunch) instead of registering the amount of the separate foods contained in one meal (i.e., 100g of chicken, 100g of lettuce, and 30 g of Caesar dressing). This introduces additional measurement errors resulting in a less accurate reference method. Lastly, a paper-based FFQ was used in 2013, while a web-based version was used in the 2019 and 2021 validation studies. However, the research by Lai et al. and Al-Shaar et al. shows that paper-based versus web-based FFQs show comparable results (36, 37).

### 4.6. Conclusion

In conclusion, the current FFQ shows low validity for estimating absolute nutrient intake but acceptable validity for estimating nutrient density intake and food group intake. Moreover, the reproducibility of the FFQ is good and misclassification is low. Therefore, it is recommended to use the FFQ only to compare nutrient density intakes or food group intake of populations and should not be used to measure actual or absolute intake. The length of the FFQ is rather short which comprises its validity. Nevertheless, this does not outweigh its great feasibility for the researcher and low burden for the participant while being a quick and low-cost dietary assessment tool that can be used on a larger scale.

## Data availability statement

The raw data supporting the conclusions of this article will be made available by the authors, without undue reservation.

## Ethics statement

The studies involving human participants were reviewed and approved by Social and Societal Ethics Committee and Medical Ethical Committee UZ Leuven. The patients/participants provided their written informed consent to participate in this study. Written informed consent was obtained from the individual(s) for the publication of any potentially identifiable images or data included in this article.

## Author contributions

JV wrote the manuscript, analyzed and interpreted the data of 2013, 2019, and 2021, and set up the study of 2021. TB set up the study of 2019, interpreted the data of 2013, 2019, and 2021, and reviewed the manuscript. CM set up the study of 2013, 2019, and 2021, interpreted the data of 2013, 2019, and 2021, and reviewed the manuscript. All authors contributed to the article and approved the submitted version.
