# Supplementary material for: Development and validity of a short web-based semi-quantitative Food Frequency Questionnaire applicable in both clinical and research setting: an evolution over time
Source: Front Nutr. 2023 May 17;10:1073559. doi: 10.3389/fnut.2023.1073559 (PMC10229889; doi:10.3389/fnut.2023.1073559)
Supplement: Supplementary file 1 [file Data_Sheet_1.docx]

***Supplementary Material***

Table S1 Intake of macronutrients, macronutrient densities, micronutrient and micronutrient densities obtained from the FFQ and the Food Record for the convergent validation study

Table S2 Intake of food groups (g/day) obtained from the FFQ and the Food Record for the convergent validation study

Table S3 Consumers-only analysis of relative validity of the Food Frequency Questionnaire compared to a 3-day food record

*a _difference_: Mean difference = Mean intake Food Record – Mean intake FFQ*

*b Wilcoxon signed rank test*

*c SCC: Spearman Correlation Coefficient*
